# Language Assessment in Multilingualism and Awake Neurosurgery

**DOI:** 10.3389/fnhum.2021.750013

**Published:** 2021-11-25

**Authors:** Maria De Martino, Andrea Talacchi, Rita Capasso, Annapina Mazzotta, Gabriele Miceli

**Affiliations:** ^1^Department of Political and Communication Sciences (POLICOM), University of Salerno, Fisciano, Italy; ^2^San Giovanni Addolorata Hospital, Rome, Italy; ^3^Brain Associati, Rome, Italy; ^4^Department of Neurosciences, Biomedicine and Movement Sciences, University of Verona, Verona, Italy; ^5^Center for Mind/Brain Sciences, University of Trento, Trento, Italy

**Keywords:** multilingualism, language assessment, brain tumor, awake surgery, intraoperative testing

## Abstract

Multilingualism has become a worldwide phenomenon that poses critical issues about the language assessment in patients undergoing awake neurosurgery in eloquent brain areas. The accuracy and sensitivity of multilingual perioperative language assessment procedures is crucial for a number of reasons: they should be appropriate to detect deficits in each of the languages spoken by the patient; they should be suitable to identify language-specific cortical regions; they should ensure that each of the languages of a multilingual patient is tested at an adequate and comparable level of difficulty. In clinical practice, a patient-tailored approach is generally preferred. This is a necessary compromise since it is impossible to predict all the possible language combinations spoken by individuals and thus the availability of standardized testing batteries is a potentially unattainable goal. On the other hand, this leads to high inconsistency in how different neurosurgical teams manage the linguistic features that determine similarity or distance between the languages spoken by the patient and that may constrain the neuroanatomical substrate of each language. The manuscript reviews the perioperative language assessment methodologies adopted in awake surgery studies on multilingual patients with brain tumor published from 1991 to 2021 and addresses the following issues: (1) The language selected for the general neuropsychological assessment of the patient. (2) The procedures adopted to assess the dimensions that may constrain language organization in multilingual speakers: age and type of acquisition, exposure, proficiency, and use of the different languages. (3) The type of preoperative language assessment used for all the languages spoken by the patient. (4) The linguistic tasks selected in the intraoperative setting. The reviewed data show a great heterogeneity in the perioperative clinical workup with multilingual patients. The only exception is the task used during language mapping, as the picture naming task is highly preferred. The review highlights that an objective and accurate description of both the linguistic profile of multilingual patients and the specific properties of the languages under scrutiny can profitably support clinical management and decision making in multilingual awake neurosurgery settings.

## Introduction

In a broad and inclusive sense multilingualism can be defined as the acquisition and use in everyday life of two or more languages (Butler, 2013; Grosjean, 2013).^1^ In order to cope with challenges resulting from migration and globalization, current human societies support multilingualism since it promotes education, cognitive health (Antoniou and Wright, 2017; Baumgart and Billick, 2018; Calabria et al., 2020), cultural, social, and economic inclusion (Aronin and Singleton, 2008). Worldwide, multilingual people are actually the rule rather than the exception, mostly if one considers that, beyond the official and standardized languages, many people use dialect for communication in everyday life (Grosjean, 1992; EuroStat, 2015; Hartsuiker et al., 2016). However, the monolingual brain and the monolingual language processing system are still considered as the norm both in neurocognitive models of language and in clinical practice. This is probably due to mixed and inconsistent findings and to several extant controversies on the functioning, architecture, and neural underpinnings of language processing in multilinguals.

Starting from late ’70, aphasiology, neurosurgery, and neuroimaging studies have provided evidence about the multilingual brain (Albert and Obler, 1978; Ojemann and Whitaker, 1978; Paradis, 2004; Bhatia and Ritchie, 2006). Two major issues have been addressed:

(1). Whether multilingual speakers recruit the same regions as monolinguals during linguistic tasks or multilingualism requires recruiting additional brain regions.
(2). Whether or not different languages require the support of specific cortical regions.

In general, clinical observations on multilingual aphasic individuals documented different patterns of impairment and of post-insult recovery in each of the languages spoken by the patients and described complex correlations between language and brain sites (Albert and Obler, 1978; Aglioti and Fabbro, 1993; Paradis, 2000; Giussani et al., 2007). Consistent findings were reported in neurosurgical settings where multilingual patients showed language-specific^2^ responses to brain stimulation (see Połczyñska and Bookheimer, 2020 for a recent review). These data have often been used as evidence that different languages are represented in different brain regions. However, neuroimaging studies in healthy multilinguals provided evidence that the neural representation of L1 converges with that of additional spoken languages (Green, 2003; Abutalebi and Green, 2007; García-Pentón et al., 2016). The contrasting results that emerge from studies on brain-damaged patients and from neuroimaging investigations on neurotypical individuals do not yet have a straightforward explanation. Reliable accounts will require substantial progress in at least two areas of investigation. In the first place, a finer-grained knowledge of the neural representation of linguistic knowledge and domain-general resources is mandatory. So far studies focused on single-word processing (mostly nouns) but largely steered clear of language-specific aspects of syntax and morphosyntax, and of their interactions with processing resources. Obviously, studies should be carried out in more languages than currently available. Secondly, results should be interpreted based on an in-depth knowledge of experimental methods. To mention but one issue, neuroimaging investigations analyze BOLD signal changes in macroareas regions of interest (ROIs) during exposure to a relatively large number of stimuli, whereas direct electrical stimulation (DES) is delivered over very small areas of the brain, each of which may occupy a minimal fraction of said ROIs, and results are inferred based on a necessarily limited number of stimuli.

At present, despite the growing amount of evidence, it is hard to draw clear and firm conclusions about the neural and cognitive organization of multilingualism. As a matter of fact, multilingualism poses a number of critical questions on both theoretical and methodological grounds. The linguistic profiles of multilingual speakers are very heterogeneous, since a multitude of experience-related factors determines the multilingual competence: age (early vs. late) and type of acquisition (formal vs. informal education; simultaneous vs. sequential acquisition), amount of exposure to the different languages, modality (oral vs. written or both) and context (familiar vs. professional or both) of use, proficiency level, and degree of similarity/distance between languages. Recent reviews and meta-analyses (Cargnelutti et al., 2019; Połczyñska and Bookheimer, 2020) have shown that those factors affect the performance of multilinguals in linguistic tasks and have an impact on the neural organization of languages. In addition, the interaction among the spoken languages has been shown to modulate their neural underpinnings (De Bot, 2004; Kroll and Bialystok, 2013; Kroll and Ma, 2018; Del Maschio and Abutalebi, 2019). However, there are no standardized objective measures or procedures to operationalize these variables. From a research/academic perspective, this is conducive to results that are not comparable across studies and thus hampers an adequate comprehension of the multilingual system and of its cerebral organization. From a clinical standpoint, a potential underestimation of the role of those factors during language assessment may produce skewed profiles of the pattern of compromised/preserved linguistic abilities in multilingual patients. For instance, an incorrect estimation of the proficiency or of the frequency of usage of the languages spoken by a multilingual speaker may produce confounds when assessing the presence of linguistic deficits. In other words, it is crucial to distinguish a true anomia, or a true semantic/phonological paraphasia, from errors due to inaccurate knowledge or infrequent use of a given language. Similarly, increased latencies in reading or naming tasks could indicate either difficulties in lexical access, or cross-language interference, or even a language-switching cost in speakers with strong dominance of one language over the others. Such a problem has relevant consequences especially for the procedures adopted during awake glioma surgery in language-sensitive brain regions. The surgical procedure with the patient in awake state has been introduced in brain tumor treatment in the ‘90s and requires the patient to perform cognitive tasks while specific parts of the brain are stimulated. If stimulation interferes with the task, the stimulated area should not be resected to prevent post-surgery deficits. This technique offers two primary advantages: it allows enrolling in surgical treatment patients who were previously excluded because their tumors were located in brain areas critical for specific cognitive functions, such as language; furthermore, it preserves full functionality while allowing maximal resection of pathological tissue. The potential variation of both linguistic competence and anatomical differences in the cortical representation of the different languages in multilingual patients may induce additional post-surgical deficits if all the languages are not comprehensively and adequately assessed preoperatively and if an appropriate intraoperative testing has not been prepared.

This manuscript analyses the perioperative language assessments adopted in awake surgery studies on brain tumors in multilingual patients published from 1991 to 2021. Albeit awake surgery procedures are usually adopted to treat severe epilepsy while preserving language functions, we focused only on brain tumor surgery. The reason is that epilepsy frequently has a childhood onset and may affect language acquisition, thus adding a possible confound in the analysis of language assessment procedures in multilingual speakers. The aim of the present manuscript is to verify to what extent the variables that affect linguistic processing in multilingual speakers have been considered during planning and decision making in awake surgery for brain tumors. The following main issues will be addressed: whether and how AoA and proficiency in each language are evaluated; how language skills are assessed in all the languages; whether the distance/similarity across languages is kept under control in the direct comparison of language-specific performance accuracy; how the language for the global neuropsychological assessment of the multilingual patient is selected and which tasks are used in the intraoperative setting. In the following paragraphs, each of the variables analyzed in the manuscript is described. Results are summarized in separate sections. Finally, strengths and weaknesses of the most frequently used clinical approaches to multilingualism in awake surgery for brain tumors are discussed.

## Materials and Methods

A literature search using PubMed and Web of Science databases was performed between March and May 2021. The following terms were used: plurilingual*, multilingual*, bilingual*, trilingual*, quadrilingual*, polyglot, brain tumor, brain tumour, brain cancer, cerebral cancer, glioma, glioblastoma, meningioma, awake surgery, craniotomy, neurosurgery, direct electrical stimulation, and electrocorticography. The asterisk indicates that terms used to enter the bibliographic databases were abbreviated. This allowed us to broaden the search by finding words that started with the same letters. Manuscripts published between 1991 and 2021 were considered.

**FIGURE 1:**
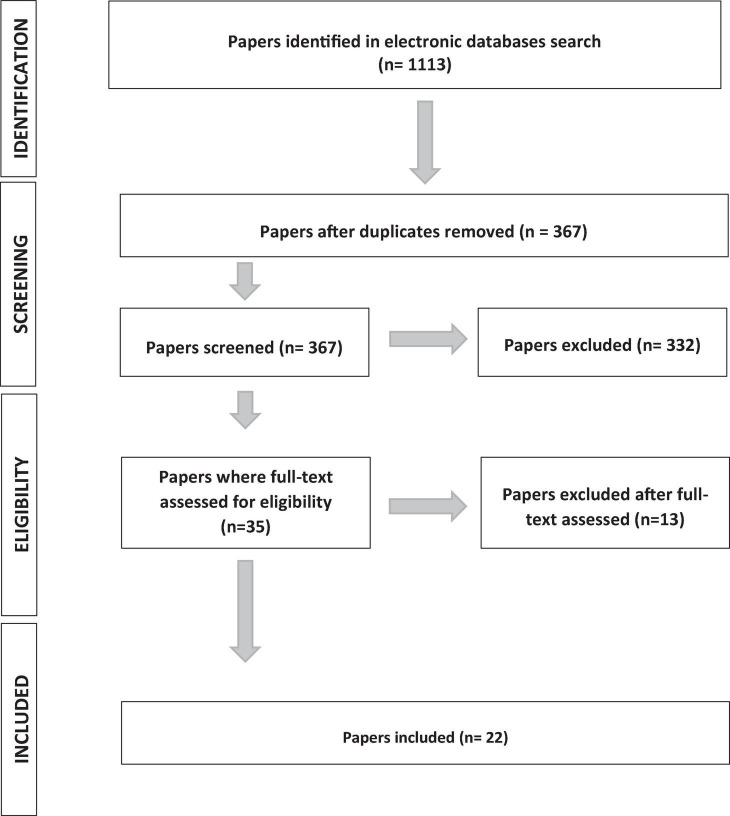
Flowchart of the search process. Numbers show how many studies were included at each stage.

We found 1,113 peer-reviewed manuscripts, removed duplications (746 manuscripts) and among the remaining 367 manuscripts we only focused on those that included multilingual individuals who underwent awake surgery. A flowchart of the research strategy is reported in Figure 1.

The following exclusion criteria were applied: review manuscript, sign languages, not in English, not a manuscript, not brain tumor, not multilingual, not adult, not intraoperative language mapping in awake surgery, not multilingual intraoperative testing, insufficient details about the preoperative and/or the intraoperative multilingual language testing.

Ultimately, 22 manuscripts were selected and reviewed. The list of the selected manuscripts is reported in the first column on the left of Tables 1–6.

**TABLE 1:** Patient information*.

Study	Number of patients	Etiology			Lesion location			Age	Handedness			Sex	
		Glioma	Metastasis	Other	RH	LH	Other		R	L	AD	M	F
Pouratian et al., 2000	1	1	–	–	–	1: Perysilvian cortices	–	43	1	–	–	–	1
Roux and Trémoulet, 2002	12	5	5	2	–	12	–	30–74	12	–	–	8	4
Lubrano et al., 2004	2	NA	NA	NA	1: Frontal	1: Frontal	–	1: 471: 67	2	–	–	1	1
Roux et al., 2004	19	NA	NA	NA	5	14	–	13–76	18	1	–	NA	NA
Walker et al., 2004	17	17	–	–	–	Precentral gyrus Central sulcus Postcentral gyrus Frontal operculum Angular gyrus	–	15–57	14	–	3	11	6
Bello et al., 2006	7	7	–	–	–	Frontal	–	32–58	7	–	–	4	3
Bilotta et al., 2011	1	1	–	–	–	Wernicke area	–	54	NA	NA	NA	–	1
Cervenka et al., 2011	1	–	–	1	–	Hippocampus	–	28	3	–	1	1	–
Borius et al., 2012	7	5	1	1	2: Superior and middle frontal gyri	1: Inferior frontal and superior temporal gyri 1: Superior, middle and inferior frontal gyri 1: Supramarginal and superior temporal gyri 1: Supramarginal gyrus 1: Supramarginal, superior temporal and inferior frontal gyri	–	26–45	5	2	–	3	4
Lubrano et al., 2012	1	1	–	–	–	Prefrontal	–	31	1	–	–	–	1
Kin et al., 2013	1	–	–	1	–	Temporal	–	40	1	–	–	1	–
Sierpowska et al., 2013	2	–	–	2	–	1: Fronto-opercular 1: Temporal	–	1: 60 1: 36	2	–	–	2	–
Wang et al., 2013	1	1	–	–	–	Frontal	–	25	1	–	–	–	1
Gao et al., 2015	11	11	–	–	–	11	–	24–46	11	–	–	8*^i^*	3*^i^*
Gao et al., 2016	6	1	–	5	–	1: Parietal 2: Temporal 1: Fronto-temporal 1: Temporal-occipital 1: Temporal	–	21–34	6	–	–	4	2
Połczyñska et al., 2016	1	–	–	1	–	Frontal	–	60	1	–	–	–	1
Fernández-Coello et al., 2017	13	8	1	4	–	13: Perisylvian language region	–	25–62	13	–	–	5	8
Sierpowska et al., 2018	7	3	–	4	–	2: Frontal 3: Fronto- temporal 2: Fronto-temporo-parietal	–	33–54	NA	–	–	5	2
Chan et al., 2019	1	1	–	–	–	Inferior frontal gyrus	–	28	1	–	–	1	–
Jain et al., 2019	1	–	–	1	Frontal	–	–	60	NA	–	–	–	1
de Macêdo Filho et al., 2020	1	–	–	1	–	Frontal	–	45	NA	–	–	1	–
ReFaey et al., 2020	14	2	–	12	–	9: Frontal^ii^ 8: Parietal 5: Temporal	–	45.2	NA	NA	NA	10	4

*RH, right hemisphere; LH, left hemisphere; R, right; L, left; AD, ambidextrous; M, male; F, female; NA, not available.

*^i:^We report here a verbatim quote: “All 11 patients were native to Guangdong, and included eight males and four females aged from 24 to 46 (mean 28.6) years.” Since 8 + 4 = 12, here we assumed that there were 8 males and 3 females.*

*^ii^Tumor may be located in overlapping eloquent regions.*

The following data were extracted from the reviewed manuscripts: demographic and clinical information about patients^3^ (Table 1), languages (number and types of studied languages, multilingual profiles and language distance/similarity, Table 2), AoA (Table 3), proficiency (Table 4), language assessment and intraoperative tasks (Table 5), and general neuropsychological assessment (Table 6).

**TABLE 2:** Number and types of studied languages and language distance/similarity.

Study	Languages					Language distance
	L1	L2	L3	L4	L5	
Pouratian et al., 2000	1: Spanish	1: English	–	–	–	Not considered
Roux and Trémoulet, 2002	12: French	6: English 2: Spanish 4: Occitan	1: German	1: Mandarin Chinese	–	*Post hoc* considerations on the role of different writing systems and on the opposition between Romance vs. Slavic vs. Asiatic languages
Lubrano et al., 2004	1: Arabic1: English	2: French	–	–	–	*Post hoc* considerations on the role of alphabetic vs. ideographic writing systems
Roux et al., 2004	19: French	8: English3: Spanish5: Occitan2: German1: Arabic	1: German;1: Russian	1: Mandarin Chinese	–	Not considered
Walker et al., 2004	1: Chinese3: Spanish1: Punjabi1: Turkish5: English1: Norwegian1: Portuguese1: Korean1: Russian1: Tagalog1: Slovenian	4: Spanish11: English1: Tagalog1: French	1: English	–	–	Not considered
Bello et al., 2006	1: Dutch1: English1: French1: Czech1: Korean1: Italian1: Arabic	2: English1: French3: Italian1: Spanish	2: French2: Italian3: English	1: Italian1: German1: Spanish1: Hungarian1: French	2: German	Not considered
Bilotta et al., 2011	English	Italian	–	–	–	Not considered
Cervenka et al., 2011	Igbo	English	–	–	–	*Post hoc* considerations about differences between language families and about differences in prosody, syntax and phonology across languages
Borius et al., 2012	4: French1: Italian1: Arabic1: Kinyarwanda	4: English2: French1: German	–	–	–	Not considered
Lubrano et al., 2012	German	English	French	–	–	Not considered
Kin et al., 2013	Japanese	English	–	–	–	Not considered
Sierpowska et al., 2013	1: Catalan1: Spanish	1: Spanish1: Catalan	–	–	–	Cognate words not included in the intraoperative task
Wang et al., 2013	Chinese	English	–	–	–	Not considered
Gao et al., 2015	11: MandarinChinese	11: CantoneseChinese	–	–	–	Not considered
Gao et al., 2016	6: Chinese	6: English	–	–	–	Not considered
Połczyñska et al., 2016	Swiss German	French	English	German	–	Language similarity (Swiss-German vs. German) was not associated to similarity in neural representation
Fernández-Coello et al., 2017	4: Catalan6: Spanish3: German	6: Spanish4: Catalan2: Basque1: French	9: English1: Catalan2: French1: Spanish	2: French1: Galician1: Russian	1: English	Description of the languages based on the distinction between Romance, Germanic, Slavic and isolate languages
Sierpowska et al., 2018	3: Spanish4: Catalan	3: Catalan4: Spanish	–	–	–	Cognate words not included in the intraoperative task
Chan et al., 2019	Tamil	English	Malay	–	–	*Post hoc* considerations about the diglossic nature of Tamil language
Jain et al., 2019	Hindi	English	–	–	–	*Post hoc* discussion about possible influence of writing systems (English vs. Hindi) on the RH involvement during intraoperative testing
de Macêdo Filho et al., 2020	Portuguese	English	–	–	–	Not considered
ReFaey et al., 2020	Not specified	Not specified	–	–	–	Not considered

**TABLE 3:** Age of acquisition*.

Study	AoA modality of assessment	AoA			
		L2	L3	L4	L5
Pouratian et al., 2000	Patient report	6 years	–	–	–
Roux and Trémoulet, 2002	Qualitative system of classification that collapses AoA, proficiency, and frequency of usage	4: before 7 years8: after 7 years	1: after 7 years	1: after 7 years	–
Lubrano et al., 2004	NA	NA	NA	–	–
Roux et al., 2004	Qualitative system of classification that collapses AoA, proficiency, and frequency of usage	NA	NA	NA	–
Walker et al., 2004	Patient report	3: before 5 years6: 5 years1: 6 years1: 8 years1:10 years2: 13 years1: 14 years1: 26 years1: 29 years	1: 6 years	–	–
Bello et al., 2006	Patient and family report	NA	NA	NA	NA
Bilotta et al., 2011	Patient report	after 18 years	–	–	–
Cervenka et al., 2011	Patient report	14 years	–	–	–
Borius et al., 2012	Beginning of L2 formal education	3: 11 years1: 6 years1: 3 years1: 4 years1: 5 years	–	–	–
Lubrano et al., 2012	Beginning of L2 and L3 formal education	10 years	12 years	–	–
Kin et al., 2013	Patient report	25 years	–	–	–
Sierpowska et al., 2013	Patient report	1: 7 years2: 14 years	–	–	–
Wang et al., 2013	Bilingual history questionnaire	13 years	–	–	–
Gao et al., 2015	Patient report	<5 years	–	–	–
Gao et al., 2016	Patient report	6: after 5 years	–	–	–
Połczyñska et al., 2016	Patient report	5 years	15 years	16 years	-
Fernández-Coello et al., 2017	Patient report	11: before 7 years2: after 7 years	13: after 7 years	3: after 7 years	1: after 7 years
Sierpowska et al., 2018	Patient report	3: before years4: after 7 years	–	–	–
Chan et al., 2019	Patient report	School age	Adulthood	–	–
Jain et al., 2019	Patient report	Childhood	–	–	–
de Macêdo Filho et al., 2020	Patient report	Adulthood	–	–	–
ReFaey et al., 2020	Patient report	14: after 6 years	–	–	–

*NA, not available.

**TABLE 4:** Language proficiency.

Study	Proficiency modality of assessment	Proficiency/amount of use/context of use				
		L1	L2	L3	L4	L5
Pouratian et al., 2000	Patient report	NA/15 times per month/family and work	NA/daily from 25 years/family and work	–	–	–
Roux and Trémoulet, 2002	Qualitative system of classification that collapses AoA, proficiency, and frequency of usage	12: high proficiency/spoken daily/NA	7: high proficiency/spoken daily/NA5: low proficiency/not spoken every day/NA	1: low proficiency/not spoken every day/NA	1: low proficiency/not spoken every day/NA	-
Lubrano et al., 2004	NA	high proficiency/NA/NA	high proficiency/NA/NA	–	–	–
Roux et al., 2004	Qualitative system of classification that collapses AoA, proficiency, and frequency of usage	NA	NA	NA	NA	–
Walker et al., 2004	% of correct responses in a 64 item object naming task	>85%/NA/NA	> 85%/NA/NA	>85%/NA/NA	–	–
Bello et al., 2006	Formal testing	score > 80% in all the tests/NA/NA	score > 80% in all the tests/NA/NA	score > 80% in all the tests/NA/NA	score > 80% in all the tests/NA/NA	score > 80% in all the tests/NA/NA
Bilotta et al., 2011	NA	NA	NA	–	–	–
Cervenka et al., 2011	Patient report	NA/NA/family life	NA/NA/family life	–	–	–
Borius et al., 2012	Patient report	high proficiency/NA/translation activity on a daily base/work activity	high proficiency: fluent in their L2 for at least 14 years/translation activity on a daily base/work activity	–	–	–
Lubrano et al., 2012	Formal linguistic testing and patient report	high proficiency/only with a few friends/family life	NA/not used in the past 13 years/NA	high proficiency/daily/work and family life	–	–
Kin et al., 2013	Patient report	NA/daily spoken/NA	NA/daily spoken/NA	–	–	–
Sierpowska et al., 2013	Self-rated skills in comprehension, reading, speaking, and writing on a 4-point scaleLanguage use assessed on a 7-point scale	1:4/predominant use/NA2: 4/predominant use/NA	1: 3.5/less frequently used/NA2:4/less frequently used/NA	–	–	–
Wang et al., 2013	National College English Test for L2; Self-rating of L2 reading, writing, speaking and listening skills	high proficiency/NA/NA	high proficiency/NA/NA	high proficiency/NA/NA	–	–
Gao et al., 2015	Patient report	high proficiency/NA/NA	high proficiency/NA/NA	–	–	–
Gao et al., 2016	National College English Test for English as L2,	high proficiency/daily use/work and family life	level 6 in the NCET but notproficiency as L1/frequently/work and study	–	–	–
Połczyñska et al., 2016	Patient report	high proficiency/frequently used/family life	high proficiency/frequently used/family life	high proficiency/frequently used/work and family life	high proficiency	–
Fernández-Coello et al., 2017	Score at a modified version of the Boston Naming TestPatient report	high proficiency/routinely used/NA	high proficiency/routinely used/NA	high proficiency/routinely used/NA	high proficiency/routinely used/NA	high proficiency/routinely used/NA
Sierpowska et al., 2018	Self-reported measures	high proficiency/NA/NA	high proficiency/NA/NA	–	–	–
Chan et al., 2019	Patient report	NA/NA/family life	high proficiency/daily basis/spoken with other none Tamil-speaking individuals	NA/NA/work life	–	–
Jain et al., 2019	Self-rating on a 10 points scale	8–9/10	4/10	–	–	–
de Macêdo Filho et al., 2020	NA	NA	NA	–	–	–
ReFaey et al., 2020	Patient self-report (speaker’s point of view)Certified translator evaluation (listener’s point of view)	NA	NA	–	–	–

**TABLE 5:** Language assessment and Intraoperative tasks.

Study	Language assessment					Intraoperative task
	L1	L2	L3	L4	L5	
Pouratian et al., 2000	Extensive language testing reported but NA	Extensive language testing reported but NA	-	-	-	Naming objects only in L2 during DES + 8 trials in L1 all languages during optical imaging
Roux and Trémoulet, 2002	Written and oral comprehensionNamingVerbal fluencyReadingCalculationDictationRepetitionWritten transcriptionObject handling	Written and oral comprehensionNamingVerbal fluencyReadingCalculationDictationRepetitionWritten transcriptionObject handling	Written and oral comprehensionNamingVerbal fluencyReadingCalculationDictationRepetitionWritten transcriptionObject handling	Written and oral comprehensionNamingVerbal fluencyReadingCalculationDictationRepetitionWritten transcriptionObject handling	-	Counting, all languagesNaming objects (This is a…), all languagesReading (sentences), all languages
Lubrano et al., 2004	Written and oral comprehensionNamingVerbal fluencyReadingDictationRepetitionWritten transcriptionCalculationObject handling	Written and oral comprehensionNamingVerbal fluencyReadingDictationRepetitionWrittentranscriptionCalculationObject handling	-	-	-	Naming (This is a…), all languagesReading (sentences), all languagesWriting (dictated text), all languages
Roux et al., 2004	Written and oral comprehensionNamingVerbal fluencyReadingDictationRepetitionWritten transcriptionCalculationObject handling	Written and oral comprehensionNamingVerbal fluencyReadingDictationRepetitionWritten transcriptionCalculationObject handling	Written and oral comprehensionNamingVerbal fluencyReadingDictationRepetitionWritten transcriptionCalculationObject handling	Written and oral comprehensionNamingVerbal fluencyReadingDictationRepetitionWritten transcriptionCalculationObject handling	-	Naming 30 objects (This is a…), all languagesReading (sentences, 30 items), all languages
Walker et al., 2004	Naming	Naming	Naming	-	-	Naming objects (single words), all languages
Bello et al., 2006	Spontaneous speechVerbal fluencyNaming (famous faces, objects, actions)Word comprehensionSentence comprehensionTranscoding tasksToken testDigit spanCounting	Spontaneous speechVerbal fluencyNaming (famous faces, objects, actions)Word comprehensionSentence comprehensionTranscoding tasksToken test Digit spanCounting	Spontaneous speechVerbal fluencyNaming (famous faces, objects, actions)Word comprehensionSentence comprehensionTranscoding tasksToken test Digit spanCounting	Spontaneous speechVerbal fluencyNaming (famous faces, objects, actions)Word comprehensionSentence comprehensionTranscoding tasksToken testDigit spanCounting	Spontaneous speechVerbal fluencyNaming (famous faces, objects, actions)Word comprehensionSentence comprehensionTranscoding tasksToken testDigit spanCounting	Namingobjects, actions and famous people (30 items), all languages
Bilotta et al., 2011	NA	NA	-	-	-	Counting, all languagesNaming objects, actions and famous people (30 items), all languages
Cervenka et al., 2011	NA	Verbal fluencySpontaneous SpeechWritingToken Test	-	-	-	Naming objects (40 item with EMS; 85 items ECoG), all languages
Borius et al., 2012	Written and oral comprehensionNamingVerbal fluencyReading (words, non-words, sentences)CalculationDictationRepetitionWritten transcriptionObject handlingTranslation (from L2 to L1)Comprehension of oral spellingWord recognitionWord-picture matchingSymbol discrimination	Written and oral comprehensionNamingVerbal fluencyReading (words, non-words, sentences)CalculationDictationRepetitionWritten transcriptionObject handlingTranslation (from L2 to L1)Comprehension of oral spellingWord recognitionWord-picture matchingSymbol discrimination	-	-	-	Naming objects, all languagesReading (sentences), all languagesTranslating form L2 to L1
Lubrano et al., 2012	Naming (nouns, verbs)Repetition (words, sentences)Narrative speechDefinition of metaphorsSemantic categories (judgment, justification)Linguistic prosody (comprehension, repetition)Emotional prosody (comprehension, repetition)Indirect speech acts (interpretation)Verbal fluencyConversation	Not assessed	Naming (nouns, verbs)Repetition (words, sentences)Narrative speechDefinition of metaphorsSemantic categories (judgment, justification)Linguistic prosody (comprehension, repetition)Emotional prosody (comprehension, repetition)Indirect speech acts (interpretation)Verbal fluencyConversation	-	-	Naming, L1 and L3
Kin et al., 2013	Auditory comprehensionNamingSentence repetitionReading aloud short sentencesReading for comprehensionDictation of Kana lettersDictation of short sentences	NA	-	-	-	Naming, all languagesAuditory responsive-naming task, all languages
Sierpowska et al., 2013	*Only Spanish language was tested:*NamingVerbal fluencyToken TestNon-words repetitionBilingual Switching Questionnaire	*Only Spanish language was tested:*NamingVerbal fluencyToken TestNon-words repetitionBilingual Switching Questionnaire	-	-	-	Naming, all languagesLanguage switching naming (40 items)
Wang et al., 2013	NA	NA	-	-	-	Naming, all languagesLanguage switching task a: Naming objects, a cue indicated the language to be usedLanguage switching task b: a cue indicated if the color or the shape of objects had to be named
Gao et al., 2015	Counting from 1 to 100NamingWord reading	Counting from 1 to 100NamingWord reading	-	-	-	Counting (from 1 to 10), all the languagesNaming (This is a…), all languagesReading (words), all languages
Gao et al., 2016	Counting from 1 to 100Naming	Counting from 1 to 100Naming	-	-	-	Counting, all languages;Naming, all languages;Word reading, all languages
Połczyñska et al., 2016	Naming	Naming	Visual namingAuditory namingVerbal fluencyRepetitionReading (word and non-word)	Naming	-	Naming objects, all languages
Fernández-Coello et al., 2017	Expressive languageNamingToken Test (brief version)Automatic languageVerbal fluencyReading taskNon-word repetitionVocabulary	Expressive languageNamingToken Test (brief version)Automatic languageVerbal fluencyReading taskNon-word repetitionVocabulary	Expressive languageNamingToken Test (brief version)Automatic languageVerbal fluencyReading taskNon-word repetitionVocabulary	Expressive languageNamingToken Test (brief version)Automatic languageVerbal fluencyReading taskNon-word repetitionVocabulary	Expressive languageNamingToken Test (brief version)Automatic languageVerbalFluencyReading taskNon-word repetitionVocabulary	Naming, all languages
Sierpowska et al., 2018	NamingComprehensionNon-words repetitionStroop testVerbal fluency The Hayling testBilingual Switching Questionnaire	NamingStroop test	-	-	-	Naming, all languagesLanguage switching naming
Chan et al., 2019	Naming	CountingNamingSemantic Association	Naming	-	-	Counting, all languagesNaming, all languagesPyramids and palm trees test, all languages
Jain et al., 2019	Bilingual Aphasia TestNaming	Bilingual Aphasia TestNaming	-	-	-	Counting, all languagesNaming, all languagesReading the mind in the eyes Test (attempted but not completed)
de Macêdo Filho et al., 2020	NA	NA	-	-	-	Naming, all languagesPyramids and palm trees test, in L1
ReFaey et al., 2020	NA	NA	-	-	-	Object naming, all languagesNon-word repetition, all languagesWord comprehension, all languages

**TABLE 6:** Preoperative and postoperative neuropsychological assessment and preoperative language mapping*.

Study	Language used for neuropsychological assessment	Preoperative neuropsychological assessment	Postoperative neuropsychological assessment	Preoperative language mapping
Pouratian et al., 2000	NA	NA	NA	fMRI in L2
Roux and Trémoulet, 2002	NA	EHI	Language assessment	NA
Lubrano et al., 2004	NA	EHI	Language assessment	NA
Roux et al., 2004	NA	EHI	Language assessment	NA
Walker et al., 2004	NA	NA	NA	WADA test for lateralization in two patients
Bello et al., 2006	NA	EHIIdeomotor apraxiaFace apraxiaDigit span	NA	fMRI in some cases
Bilotta et al., 2011	NA	NA	NA	NA
Cervenka et al., 2011	English (L2), language most frequently used and where normative data were available	WAIS-RRAVL test	NA	NA
Borius et al., 2012	NA	NA	Language assessment	NA
Lubrano et al., 2012	NA	EHIFigure Copying (2 intersecting pentagons)Clock drawing	Language assessment	NA
Kin et al., 2013	NA	MMSEFAB	Language assessment (L1)	NA
Sierpowska et al., 2013	Spanish, language where normative measures were available	EHIDigit span	Language assessmentDigit span	NA
Wang et al., 2013	NA	KPS	NA	NA
Gao et al., 2015	Mandarin Chinese (L1) and Cantonese Chinese, (L2)	EHIMOCA	MOCALanguage assessment	NA
Gao et al., 2016	NA	EHIMMSE	Language assessment	fMRI
Połczyñska et al., 2016	English (L3), language most frequently used and where normative data were available	Attention taskWorking memory taskVerbal executive ability	NA	fMRI
Fernández-Coello et al., 2017	NA	EHIDigit Span	Digit spanLanguage assessment	fMRI
Sierpowska et al., 2018	Catalan, Spanish (L1)	EHIStroop testDigit span	Stroop testDigit spanLanguage assessment	fMRI
Chan et al., 2019	English (L2), language most frequently used and where normative data were available	MOCAList learningStory memoryFigure copyLine orientationDigit spanCodingFigure recallSpatial spanBlock designStroop testColor trails testPraxis testInterlocking fingers	MOCAList learningStory memoryFigure copyLine orientationDigit spanCodingFigure recallSpatial spanBlock designStroop testColor trails testPraxis testInterlocking fingers	NA
Jain et al., 2019	Hindi (L1); English (L2)	MOCAVerbal new learningimmediate and delayed recallRME test	MOCAmore comprehensive neuropsychological testing (NA)	NA
de Macêdo Filho et al., 2020	NA	NA	Complete neurologic examination and linguistic neurocognitive assessment (tests NA)	NA
ReFaey et al., 2020	NA	KPS	KPS	fMRI

*NA, not available; EHI, Edinburgh Handedness Inventory; WAIS-R, Wechsler Adults Intelligence Scale – Revised; RAVL, Rey Auditory Verbal Learning; MMSE, Mini Mental State Examination; FAB, Frontal Assessment Battery; KPS, Karnofsky Performance Scale; MOCA, Montreal Cognitive Assessment; RME, Reading the Mind in the Eyes; fMRI, Functional Magnetic Resonance Imaging.

### Analyzed Variables

#### Language Distance

The concept of distance between languages has to do with qualitative and quantitative differences that may involve many domains: *phonetics* (tonal vs. non-tonal languages, e.g., Mandarin Chinese vs. English), *orthographic systems* (alphabetic vs. logographic, e.g., English vs. Japanese Kanji; direction of writing, e.g., right to left vs. left to right vs. top to bottom, as in Arabic vs. French vs. traditional Japanese; deep vs. transparent orthography, e.g., English vs. Italian), *vocabulary* (e.g., presence/absence of cognates^4^), *grammar* (e.g., presence/absence of determiners, grammatical gender), *morphology* (e.g., inflectional systems, agreement patterns, auxiliaries), and *syntax* (e.g., word order, phrase structure). There is no well-established method to quantify the similarity between languages and it is hard to reduce all these parameters to a single distance score (Gamallo et al., 2017). Usually, linguistic distance is determined by measuring the number of branches between two languages on the language family tree model (Dimmendaal, 1995; Połczyñska and Bookheimer, 2020). This system is based on the possibility to identify common ancestors of languages and to define broad categories of language families (e.g., Romance, Germanic, Scandinavian, African, Slavic, Semitic, Asiatic, and isolated). However, and even beyond its theoretical limitations, such an approach is scarcely useful when attempting to understand how language distance affects cognitive aspects of language processing in multilinguals. Conversely, the problem has been dealt with repeatedly in neuroscience and psycholinguistics (Vaid, 1983; Gleitman, 1985; Odlin, 1989; Cenoz et al., 2001; Koda, 2005; Bassetti, 2008; Kim et al., 2016; Schepens et al., 2016; Zawiszewski and Laka, 2020; Shinozuka et al., 2021). Models of multilingual processing have tried to define how the cognitive system manages shared and distinctive features between languages. In general, it is assumed that conceptual information on words is represented in a language-independent fashion, as it is related to the semantic properties of the word’s referents (Francis, 2005). Other aspects are controversial. On the “shared syntax” approach, syntactic-grammatical properties common to different languages are represented only once in the multilingual language system, thus reducing redundancy and increasing efficiency of language processes (Hartsuiker et al., 2004). On the other hand, the structural similarity across languages modulates the functioning of the hypothesized unified syntax (Runnqvist et al., 2013). Similarly, Peeters et al. (2013) demonstrated that identical interlingual cognates are stored as a single orthographic representation but as two distinct phonological and morphological representations, and that the activation of each representation can vary with the linguistic operation to be performed. In a study on the behavioral and neural correlates of naming in L2 in healthy speakers, Ghazi-Saidi and Ansaldo (2017) found that naming in L2 is more effortful and demanding in distant language pairs than in close language pairs.

To sum up, despite the difficulty operationalizing the distance/similarity across languages and even though the mechanisms of cerebral-cognitive assimilation and accommodation during the processing of multiple languages are still largely unknown (Kim et al., 2016), it is reasonable to conclude that specific properties of each language impose different cognitive demands on multilingual speakers. The obvious implication for researchers and clinicians is to adapt the perioperative procedures used when selecting tasks and stimuli for language assessment so as to properly address the language distance issue.

#### Age of Acquisition

The AoA generally indicates the age of exposure to a language and is taken as an indication of the moment in life when that language is acquired. The AoA parameter provides indirect information on the way a language is acquired: for instance, it can be used to make inferences about whether or not the speaker received any kind of formal education in his/her additional languages. Qualitatively different multilingual conditions have been described by using the AoA parameter (Kim et al., 1997). *Simultaneous multilingualism* applies to children who are exposed to two or more languages from birth or shortly after birth. In this case, there is no chronological gap between the first language (L1) and other languages (L2 and L3, etc.); thus, it is assumed that simultaneous multilingual speakers acquire all their languages through similar developmental trajectories and learning mechanisms. *Early-sequential multilinguals* begin to acquire additional languages after acquiring the basic grammatical structures of L1; this happens from ages 3 to 5–7 years. *Late-sequential multilinguals* acquire additional languages by the age of 5–7 years and often, albeit not always, receive formal education in these other languages (Perani et al., 2003; Leonard et al., 2011; Połczyñska et al., 2016). In the literature, different age ranges have been proposed to distinguish simultaneous, early and late bilingualism and it is plausible that AoA should be thought of as a continuous rather than a categorical parameter. Several studies demonstrated that people who learn a language in infancy generally achieve greater proficiency than late learners (Johnson and Newport, 1989; Birdsong, 1999, 2014; Perani et al., 2003), that AoA affects several language-specific skills like lexical access, phonology, grammar and syntax (Weber-Fox and Neville, 1996; Mahendra et al., 2003; Perani et al., 2003; Wartenburger et al., 2003; Frenck-Mestre et al., 2005; Hernandez et al., 2007; Isel et al., 2010; Waldron and Hernandez, 2013; Wei et al., 2015) as well as domain-general cognitive control mechanisms (Luk et al., 2011; Tao et al., 2011). Moreover, AoA has an actual role in shaping multilingual brain networks (Perani et al., 1996, 1998; Fabbro, 1998, 2001; Wartenburger et al., 2003; Mechelli et al., 2004; Abutalebi et al., 2013; Klein et al., 2014; Wei et al., 2015; Liu and Cao, 2016; Del Maschio and Abutalebi, 2019).

#### Proficiency

Proficiency indicates how well a language is known either in production or in comprehension and denotes the level of competence attained in each language (Del Maschio and Abutalebi, 2019). It is strictly related both to fluency, which refers to the speed and automaticity of linguistic behavior (Segalowitz, 2010), and to the context and amount of use and exposure to a given language. Proficiency is a multidimensional construct (Treffers-Daller, 2019), which can be differently related to specific aspects of linguistic competence such as modality (oral vs. written), task (single word/sentence or discourse production, word recognition, and language comprehension), domain (syntax, semantics, morphology, phonology, and vocabulary). Proficiency in a language can change over time: for instance, multilingual speakers can become more proficient in later-acquired languages than in their mother tongue if they stop using the latter in everyday life or only use it occasionally. For similar reasons, they can be very proficient in a specific modality, or achieve better vocabulary than grammatical skills, or vice versa.

Even if the relative relevance of the many features that define language proficiency remains unclear, the measures of proficiency used to assess the linguistic competence of multilingual speakers should be spelled out in published reports. Recent findings indicate that proficiency and frequency of use of additional languages are key factors in the organization of language networks in the multilingual brain (Stowe and Sabourin, 2005; Kotz, 2009; Consonni et al., 2013; Sugiura et al., 2015).

#### Preoperative Language Assessment

Preoperative language testing for patients undergoing awake surgery should provide detailed information on all aspects of their linguistic competence to detect aphasic deficits, identify the functional locus of damage to the language system and select the most suitable tasks/stimuli for intraoperative testing (Miceli et al., 2012). Usually, this goal is accomplished by employing standardized language batteries that provide tasks for the evaluation of different modalities (written and oral), functions (production, comprehension, transcoding, and verbal memory), and levels of language organization (materials controlled for distributional, phonological, lexical, grammatical/morphological, syntactic, and semantic features). The language assessment of multilingual patients eligible for awake surgery has specific requirements but suffers from the lack of standard procedures. Few standardized tests include multilingual materials and provide normative data from multilingual individuals (Goral and Conner, 2013; Fernández-Coello et al., 2021; Gisbert-Muñoz et al., 2021). Even when such data exist, it is practically impossible to find tests that are adequately matched in all the possible language combinations of the multilingual population^5^. In clinical settings, the standard practice consists of adapting the tests available in one of the languages spoken by a multilingual person to the other languages.

#### General Neuropsychological Assessment

Extensive neuropsychological investigations are indispensable in the clinical work-up of brain tumors. More in detail, executive functions, working memory, attention, and emotional status, at the minimum, must be assessed since they impact on linguistic performance and on the ability to tolerate the brain stimulation procedure (Talacchi et al., 2013). The preoperative assessment provides critical information about the cognitive deficits induced by the tumor so that results can be used to plan the surgical approach and define a baseline for subsequent evaluations. The postoperative assessment allows identifying the short-term and long-term outcomes of treatment and provides indications for rehabilitation (Miceli et al., 2012).

Neuropsychological assessment in multilinguals suffers from several biases. The main bias is related to the socio-cultural background of multilingual speakers, especially in immigration contexts (Ardila et al., 1994; Fortuny and Mullaney, 1997; Gasquoine, 1999). Additional biases stem from the properties of testing tools: low scores in a test obtained by a person belonging to an ethnical/linguistic group different from that in which normative data were collected, may be due to reasons other than neurological or cognitive factors (Anastasi, 1988). Potentially unpredictable biases may depend on the unique characteristics of the individual, such as the number, type, and combination of the spoken languages.

Informal testing and translated test materials are frequently used in clinical settings and may be the best possible compromise when a balance between acceptability and adequacy is warranted. However, a crucial issue concerns the selection of the language for the neuropsychological assessment. This decision is strictly related to the linguistic profile of the patient, as s/he must be able to complete the clinical interview and to understand test instructions with as little difficulty as possible. Multilinguals who are equally proficient in all their languages collaborate without difficulty during interviews and testing, as their competence is not dissimilar from that of monolingual speakers. Under the same circumstances, however, so-called functional multilinguals, who use different languages depending on the context (e.g., at work vs. in the family) may face serious difficulties. This is frequently the case of newly arrived immigrants.

#### Intraoperative Testing

Language is by far the cognitive process tested most frequently in awake surgery for brain tumors. However, intraoperative tasks and/or batteries show an extreme variability across studies. Automatic speech (e.g., counting and reciting word series) and object naming, especially adaptations of the Test de Dénomination Orale D’Images (DO80, Deloche and Hannequin, 1997) and of the Boston Naming Test (BNT, Kaplan et al., 1983), are the most commonly used tasks. Standardized tests and many other tasks are also employed (see Rofes and Miceli, 2014; Ruis, 2018; Papatzalas et al., 2021, for recent reviews). In general, intraoperative paradigms must meet specific criteria that allow both to perform an accurate and sensitive language assessment and to minimize risks in the surgical procedure. Rofes and Miceli (2014) suggested that intraoperative tasks specific for language mapping should be adapted to different kind of constraints. Some constraints are imposed by the requirements of language mapping techniques: thus, tasks should be short, allow fast stimulus-response cycles and require simple responses that can be easily scored. Other constraints depend on the language under scrutiny: relevant language-specific properties should be tapped. Furthermore, clinical constraints require that tasks and stimuli should be sufficiently sensitive as to identify fine-grained deficits, should tap specific components of the language system and should be appropriately related to the brain areas associated with the assessed language processes.

These criteria are even more stringent when multilingual people must be assessed intraoperatively.

Recently, a multilingual naming task has been standardized in nine different languages (Spanish, Basque, Catalan, Italian, French, English, German, Mandarin Chinese, and Arabic) with the specific aim to minimize linguistic distance between different groups of items. It includes colored drawings of objects and actions; stimulus words are controlled for name agreement, frequency, length, and substitution neighbors. Depending on the language combination, the test includes between 25 and 30 items and can be administered in a maximum of 5 min per language (Gisbert-Muñoz et al., 2021).

## Results

### Patient Characteristics

**FIGURE 2:**
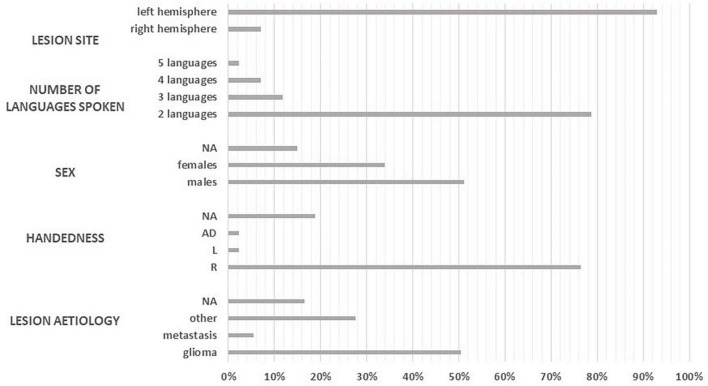
Patient information: lesion site, number of languages spoken, sex, handedness, and aetiology.

Overall, the studies reviewed here included 127 multilingual patients with brain tumors who underwent awake surgery. The histology of the tumor was reported in 93 cases (56 gliomas, 6 metastases, and 31 other tumors). Lesions were predominantly located in the left hemisphere (LH) but nine cases with right hemisphere (RH) lesions were reported (see Table 1 and Figure 2).

### Language Assessment

#### Number and Types of Studied Languages, Multilingual Profiles, and Language Distance/Similarity

The selected manuscripts investigated 31 languages and reported on different multilingual profiles. The majority of patients were bilingual (100), 15 were trilingual, 9 were quadrilingual, and 3 patients spoke five languages. A great heterogeneity in the number and type of language combinations was observed, ranging from very close (e.g., Spanish/Catalan; Mandarin Chinese/Cantonese Chinese) to very distant pairs (e.g., Arabic/French; Japanese/English).

In the reviewed manuscripts, the issue of language distance was either not considered (13 studies out of 22) or poorly addressed. A possible reason is that it is taken for granted that some language combinations have higher levels of mutual intelligibility than others and that the processing of a given language is influenced by the properties it shares with other languages (Jeong et al., 2007; Gooskens et al., 2018). In two studies (Sierpowska et al., 2013, 2018) special attention was paid to the selection of stimuli for the intraoperative task, where a possible confound due to high language similarity was avoided by excluding cognates. In one study (Fernández-Coello et al., 2017) languages were classified in different families but authors did not describe whether and how they exploited such information in surgical planning. Five studies (Roux and Trémoulet, 2002; Lubrano et al., 2004; Cervenka et al., 2011; Chan et al., 2019; Jain et al., 2019) reported only *post hoc* considerations on how across-language variations of different factors may have affected performance. One study (Połczyñska et al., 2016) explicitly investigated if language similarity (Swiss-German and German) was associated to similarity in neural representation and found this not to be the case (see Table 2).

#### Age of Acquisition

With one exception (Lubrano et al., 2004), all the selected studies reported data about the AoA of all the languages spoken by the patients (see Table 3). Most studies distinguished between early and late acquired languages but operationalized the variable differently; four studies only distinguished languages acquired during childhood vs. adulthood (Bilotta et al., 2011; Chan et al., 2019; Jain et al., 2019; de Macêdo Filho et al., 2020). The remaining studies either reported AoA or distinguished between early-acquired and late acquired multilingualism. In these cases, 7 and 5 years were the most used cut-offs between early and late AoA.

**FIGURE 3:**
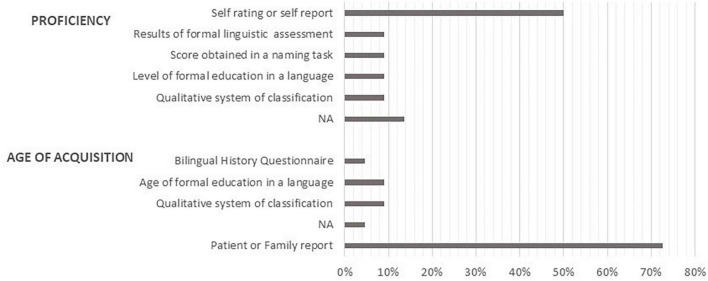
Methods used to assess proficiency and age of acquisition.

The variable was assessed differently across studies (see Figure 3). Wang et al. (2013) used a Bilingual History Questionnaire [BHQ, Li et al., 2006]. Two studies indicated the age at which patients started receiving formal education in languages other than L1 (Borius et al., 2012; Lubrano et al., 2012). Two studies (Roux and Trémoulet, 2002; Roux et al., 2004) used a qualitative classification of multilingualism where measures of AoA, proficiency and frequency of usage were collapsed into a unique score. The remaining studies (16 out of 22) collected information on AoA through patients’ or family reports.

The only study (Fernández-Coello et al., 2017) that focused specifically on how AoA influences the cortical organization of language in multilinguals undergoing awake surgery procedures for glioma resection reported more early-specific than late-specific cortical language sites, irrespective of the location of the stimulated area.

#### Proficiency

In our sample, three studies (Lubrano et al., 2004; Bilotta et al., 2011; de Macêdo Filho et al., 2020) did not report any information about how proficiency was assessed.

Two studies (Sierpowska et al., 2013; Jain et al., 2019) chose self-ratings on Likert-like scales to obtain information on language proficiency. In one case (Sierpowska et al., 2013) proficiency was evaluated also from a receptive point of view through the judgment provided by a certified translator.

Two studies (Roux and Trémoulet, 2002; Roux et al., 2004) used a qualitative measure of linguistic performance classification which collapsed AoA, proficiency and frequency of usage. In two studies (Wang et al., 2013; Gao et al., 2016) the proficiency in L2 was assessed by using the level achieved in the formal education of L2. Two studies (Walker et al., 2004; Fernández-Coello et al., 2017) used the scores obtained in naming tasks, while two (Bello et al., 2006; Lubrano et al., 2012) used the results of formal language assessment as indexes of proficiency in the different languages. The remaining nine studies used patient self-reports as the only indicator of proficiency in all the languages (see Figure 3).

Across the selected studies, additional information was occasionally provided about the amount of use/exposure and the context of use of the languages spoken by the patients. However, only qualitative information was provided, in the absence of objective measures. All these results are reported in Table 4. It is worth noting that only in one case (Lubrano et al., 2012) information on proficiency, context and amount of language use was considered in surgical planning, in order to decide which languages should be tested intraoperatively.

#### Preoperative Language Assessment

**FIGURE 4:**
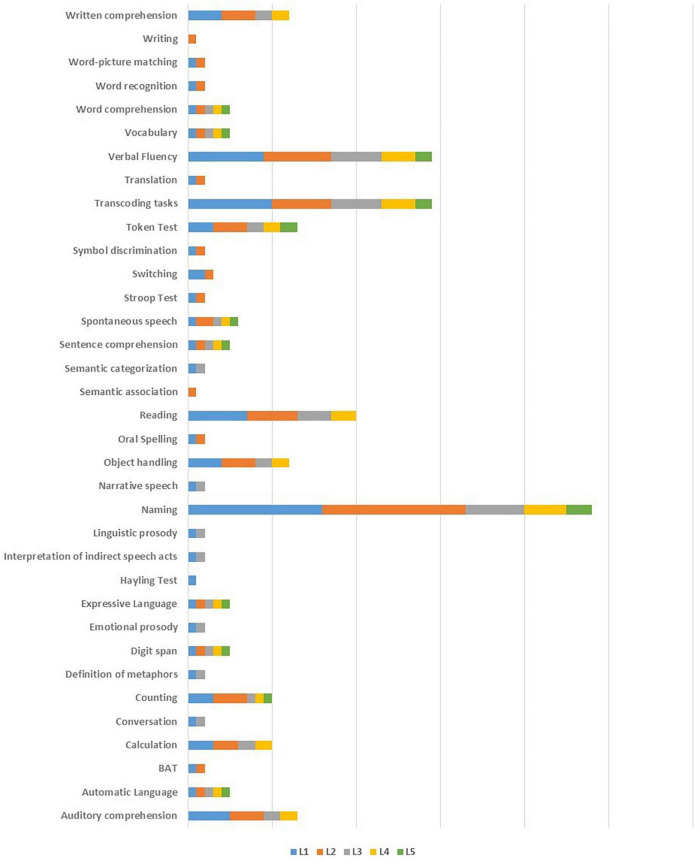
Tests used in preoperative language assessment in each language.

Of the studies considered in this manuscript, four did not describe the procedures used for language assessment (Bilotta et al., 2011; Wang et al., 2013; de Macêdo Filho et al., 2020; ReFaey et al., 2020). In one case (Pouratian et al., 2000) the authors reported that extensive testing was performed in all the languages of the patient but did not describe it. Six studies out of 22 directly compared performance accuracy in the languages spoken by the patient (Roux and Trémoulet, 2002; Lubrano et al., 2004; Roux et al., 2004; Bello et al., 2006; Borius et al., 2012; Fernández-Coello et al., 2017). In these studies, a variety of standardized tests was administered in all the relevant languages. When this was not feasible, *ad hoc* translations of the materials were used. Walker et al. (2004) used only a naming task. Połczyñska et al. (2016) assessed only one of the languages used most frequently by the patient (L3) via a composite test battery; for L1, L2, and L4 only a naming test was used. In two studies (Kin et al., 2013; Sierpowska et al., 2018) L1 was tested in a variety of tasks, while other languages were either not assessed at all (Kin et al., 2013), or were assessed only through the tasks that were going to be used intraoperatively (Sierpowska et al., 2018). In two studies (Cervenka et al., 2011; Sierpowska et al., 2013) authors only assessed the language for which standardized tests were available. In one of these studies (Cervenka et al., 2011) L1 was not assessed at all, while in the other (Sierpowska et al., 2013) it was assessed only through a picture naming task. Gao et al. (2015) tested word counting (from 1 to 100), reading aloud and naming in all the languages, while Gao et al. (2016) only tested word counting (from 1 to 100) and naming. Jain et al. (2019) administered the BAT and a naming task in the languages they studied. Chan et al. (2019) tested L2 by means of counting, naming and a semantic association task, and L1 and L2 only through a naming task that was planned to be used intraoperatively. Language assessment procedures are summarized in Table 5 and Figure 4.

In eight studies out of 22, pre-surgical language mapping methodologies were employed: Walker et al. (2004) used the WADA test to determine language laterality, while seven studies employed fMRI procedures (Pouratian et al., 2000; Bello et al., 2006; Gao et al., 2016; Połczyñska et al., 2016; Fernández-Coello et al., 2017; Sierpowska et al., 2018; ReFaey et al., 2020).

### General Neuropsychological Assessment

In our sample, three studies (Pouratian et al., 2000; Walker et al., 2004; Bilotta et al., 2011) did not report details about the general neuropsychological assessment. The remaining studies provided very heterogeneous selections of tests in both pre and postoperative assessments (see Table 6).

Seven studies provided details on pre and postoperative neuropsychological assessments (Sierpowska et al., 2013, 2018; Gao et al., 2015; Fernández-Coello et al., 2017; Chan et al., 2019; Jain et al., 2019; ReFaey et al., 2020). Four studies did not report any information on postoperative assessment (Bello et al., 2006; Cervenka et al., 2011; Wang et al., 2013; Połczyñska et al., 2016). In seven studies the postoperative assessment was restricted to language (Roux and Trémoulet, 2002; Lubrano et al., 2004; 2012; Roux et al., 2004; Borius et al., 2012; Kin et al., 2013; Gao et al., 2016). In one study (de Macêdo Filho et al., 2020) a complete neurologic examination and the language assessment were performed only after surgery but no information was provided.

The procedures adopted for preoperative assessment in the reviewed manuscripts can be thus summarized. Two studies (Wang et al., 2013; ReFaey et al., 2020) reported only the Karnofsky Performance Scale (KPS, Karnofsky and Burchenal, 1949). Three studies (Roux and Trémoulet, 2002; Lubrano et al., 2004; Roux et al., 2004) reported the Edinburgh Handedness Inventory (EHI, Oldfield, 1971). Seven studies used the EHI and additional tests for apraxia, working memory and global screening scales (Bello et al., 2006; Lubrano et al., 2012; Sierpowska et al., 2013, 2018; Gao et al., 2015, 2016; Fernández-Coello et al., 2017). Three studies provided global screening scales and a few additional tests (Cervenka et al., 2011; Kin et al., 2013; Jain et al., 2019). Połczyñska et al. (2016) assessed attention, working memory, and verbal executive abilities but did not report the employed tests. Chan et al. (2019) administered a composite battery for neuropsychological assessment.

Seven studies out of 17 provided additional information about the language selected for neuropsychological assessment: Sierpowska et al. (2018) performed neuropsychological assessment in L1; Gao et al. (2015) and Jain et al. (2019) in both L1 and L2; Cervenka et al. (2011), Sierpowska et al. (2013), Połczyñska et al. (2016), and Chan et al. (2019) used the language spoken most frequently by their patients and for which standardized tests were available.

### Intraoperative Tasks

**FIGURE 5:**
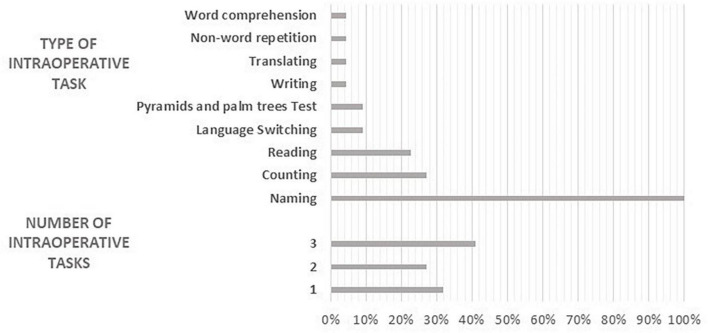
Number and type of intraoperative tasks.

A variety of tasks and of task combinations were employed for intraoperative testing in the studies reviewed here: seven studies used one task, six studies used two tasks, and nine studies used three tasks (see Table 5 and Figure 5).

A picture naming task was used in all the studies. Number and type of stimuli varied (objects, actions, and famous faces) as well as the naming context (single word vs. short sentence (“This is a…”). Six studies used a counting task (Roux and Trémoulet, 2002; Bilotta et al., 2011; Gao et al., 2015, 2016; Chan et al., 2019; Jain et al., 2019), three studies used a sentence reading task (Roux and Trémoulet, 2002; Lubrano et al., 2004; Borius et al., 2012), two studies a word reading task (Gao et al., 2015, 2016), three studies used a specific language switching task (Wang et al., 2013; Sierpowska et al., 2013, 2018), and two studies (Chan et al., 2019; de Macêdo Filho et al., 2020) used the Pyramids and Palm Trees Test (PPT, Howard and Patterson, 1992) in order to test the patient’s ability to access meaning from words. The following tasks were also employed: writing to dictation, translation form L2 to L1, naming orally described objects, word comprehension, repetition (see Table 5 for details). In addition to linguistic tasks, one study (Jain et al., 2019) included a mentalizing test in the intraoperative protocol (The reading the mind in the eyes test (RME, Baron-Cohen, 1997); however, the test was only attempted but not concluded by the patient during the surgical session.

None of the selected studies reported on how stimuli were matched across languages, nor provided information on the specialty and linguistic competence of the clinician who conducted the linguistic evaluations. The tasks described in these studies tapped different aspects of the functional architecture of language, but authors did not specify the criterion followed in task selection, except for the studies focusing on voluntary language-switching^6^ (Sierpowska et al., 2013, 2018; Wang et al., 2013).

## Discussion

This manuscript aimed at describing the state of the art in the perioperative language assessment of multilingual patients undergoing awake surgery for brain tumor. Twenty-two studies, published over the last 30 years, were reviewed. Special attention was devoted to the procedures employed to describe the patients’ multilingual profiles for their crucial role in determining the neuroanatomical organization of multiple languages and effects on cognitive functioning (Cargnelutti et al., 2019; Połczyñska and Bookheimer, 2020). Among the linguistic experience-related factors, AoA and proficiency were analyzed. Almost all the reviewed studies provided scores for those variables but assessed them differently. Noteworthy, no strong statement was reported about whether and to what extent AoA and proficiency scores helped planning intraoperative procedures (e.g., selecting languages, tasks, stimuli, and stimulation sites) nor if they had an impact on the outcome of surgery. This finding alone shows that information on AoA and proficiency has not been properly used to shed light on the cerebral organization of multiple languages. Such a bias could be neutralized if multilingual patients eligible for awake surgery were systematically questioned to obtain objective measures of their multilingualism. As for multilingualism history, the following data should be collected: AoA of L1 and of other languages; setting in which languages were acquired/learned; primary language used in school education; formal education received in each language; global amount of exposure to each language. Where and how languages are used should be ascertained through questions about context (familiar, social, and professional), linguistic profiles of interlocutors (native vs. non-native speakers), modality (spoken, written, formal, and informal), language-related media preferences (television, radio, newspaper, and internet), and frequency of use of each language in each modality in recent months.

Proficiency should be assessed preferably through subjective and objective ratings along several dimensions: proficiency in different contexts, modalities and linguistic domain, perceived accent in different languages, probability of spontaneous language switching, cross-linguistic flexibility, amount of engagement in translation activity, skills associated with effective communication, and family/friends and patient’s sense of impairment in the different languages. It is worth to underline here that all these variables related to multilingualism should be operationalized and treated comparably in awake surgery settings, in order to obtain reliable findings that could be additionally supported by formal statistical analyses in cross-linguistic studies. This might significantly improve the understanding of cerebral organization of multiple languages.

A precise and objective description of the patient’s multilingual profile should be efficiently used also for the general neuropsychological assessment. So far, seven studies reviewed in this manuscript specifically addressed this issue and reported that language used for testing was carefully chosen. Language selection depended on the availability of standardized neuropsychological tools in the language most frequently used by the patient at the moment of surgery. Such an approach should be encouraged as it prevents misinterpretation deriving from patients’ unbalanced proficiency or mastery of one language over the others, which may in turn reduce compliance with the evaluation setting, produce inaccurate comprehension of task requirements and, consequently, induce unreliable performances in neuropsychological tests (Rosselli et al., 2002; Gasquoine et al., 2007; Bender, 2015). Obviously, when the linguistic competence of the neuropsychologist is not sufficient to conduct the evaluation in the selected language, the support of (psycho)linguists and interpreters is needed. This is often difficult to afford, but it holds the obvious advantage that it allows collecting reliable information on the cognitive status of the patient.

A final aspect of the general neuropsychological assessment is worth considering. Given that the requirement of well-matched normative data is unlikely to be ever met due to the heterogeneity and variability of multilingual populations, an effective approach would consist in relying less on quantitative information (comparison of the patient’s score with that of a normative sample) and more on qualitative information about the patient’s performance in various tests.

A further crucial feature addressed in this review is the type of preoperative language assessment to be used for all the languages spoken by multilingual patients. The reviewed manuscripts showed great variability with respect to this dimension but, due to the lack of standardized multilingual tests, they did not prevent the possibility that languages may be accidentally assessed by non-equivalent modalities and at different levels of difficulty. The risk here is to underestimate or overestimate language difficulties in one language over the others, and consequently to miss the specific goal of intraoperative testing. Most studies reviewed in this manuscript suggest that, in principle, all languages should be assessed across functions (reading, writing, repetition of words and non-words, comprehension and production of words and sentences) and domains (lexicon, semantics, phonology, grammar, morphology, and syntax). In those manuscripts, the use of translated materials is common but only few details are provided about the implementation of the tasks and of the lists of stimuli.

Noteworthy, caveats should be considered. Items and tasks selected for language assessment must respect the culture standards of the languages under scrutiny; thus, culturally biased items should be avoided (Luke et al., 2002; Cheung et al., 2006). When translated from one language into another, test items should also undergo an independent back-translation in order to avoid phenomena of lexical ambiguity or synonymy. Conversely, when translation does not achieve the purpose of obtaining well-matched testing materials across different languages, additional criteria should be respected. Some examples may help clarify this point. The English verb ‘‘to knit’’ can be translated to Italian by using the multi-word expression ‘‘*lavorare a maglia.*’’ Since the two items are not equivalent on a lexical ground, they should be replaced by alternative pairs. In other words, it is not necessary to include the same items in all the languages under scrutiny but, rather, it is recommended that the words used in each language be matched for the main variables that affect linguistic processing (length, phonological complexity, frequency, AoA, imageability, grammatical class, semantic category, syntactic features, and morphological structure).^7^ This allows a good control on the cross-linguistic difficulty of tasks. Similarly, in comprehension tasks (e.g., word/picture matching or verification), the selected stimuli should be associated with appropriate semantic and phonological foils. Semantic foils usually do not suffer from translation biases, but phonological foils do. For instance, in Italian, “*sarta”* (seamstress) is a good phonological distractor for “*carta”* (manuscript); the same pair does not work when translated to English and should be changed by an equivalent pair (e.g., “boy/toy”). Again, language-specific critical features (e.g., presence/absence of case-marking, specific morphological rules, word-order constraints, and pro-drop patterns, etc.) might preclude the possibility to build perfectly matched lists of materials. For instance, the sentence “*mangio la mela*” is not fully equivalent to its English version “I eat the apple.” Italian is a pro-drop language where independent clauses may lack an explicit subject/pronoun since it is grammatically inferable by verbal inflection that, in turns, provides information on person and number. In English, an explicit subject is normally needed in sentence structure. Moreover, in Italian, in order to select the appropriate determiner for “*mela*,” speakers must retrieve information about grammatical gender and perform an operation of determiner + noun agreement while English speakers do not. This also holds for naming tasks when a minimal sentential context is required: “This is the boy,” noun + determiner (gender) agreement not required, vs. “*Questo è il ragazzo*,” noun + determiner (gender) agreement required. These few examples are a strong reminder that the specific properties of each language under evaluation should be carefully considered so that their distance and similarities are clearly and “objectively” defined. This is indispensable for a reliable language assessment in multilinguals as shared properties impact their language-specific cerebral organization (Połczyñska and Bookheimer, 2020). Nevertheless, the present review shows that this problem has been almost totally neglected in multilingual awake surgery settings.

Similar considerations hold for materials to be included in the tasks for intraoperative testing. In addition, in this latter case, the issue of how to overcome the problem of language-specific properties intersects other critical concerns. The goal of language testing during awake surgery in multilinguals is to find shared/distinct areas and networks related to different languages, so as to minimize the likelihood of postoperative (multi)linguistic disorders. On the other hand, it is necessary to find an optimal trade-off between the duration of the intraoperative testing and the neurosurgical procedure (Mandonnet et al., 2020). Thus, the number of languages to be tested and the range of intraoperative linguistic tasks must comply both with the time constraints and with the (multi)linguistic needs of the patient. The patient should be asked which language is most important to him/her and in which language(s) s/he would like to be tested during surgery. On the other hand, s/he should feel comfortable and not overwhelmed throughout the operation and should be aware of the benefits and risks of testing or not all his/her languages. In the reviewed studies, 20 surgical teams out of 22 tested all languages and agree that the ideal testing should include all the languages or at least all the most relevant spoken by the patient. However, they used different (combinations of) tasks and did not report on how they dealt with the problems linked to direct comparison between languages, language distance, and lack of standardized tools.

An accurate analysis of the linguistic behavior of the patient is crucial to optimize intraoperative procedures and, consequently, to evaluate postoperative outcomes. When standardized multilingual instruments are not available, the preparation of sufficiently specific and sensitive patient-tailored intraoperative testing should include the following steps. A picture naming task should be included, since tasks of this type have been extensively employed in awake surgery and meet the main requirements of the stimulation setting: fast presentation, easy scoring, and good patient compliance (Miceli et al., 2012; De Witte and Mariën, 2013). Nouns (objects) and finite verbs (actions) inserted in a minimal phrasal context (“This is the….”; “He/she/it ….”) should be selected as stimuli for each language. This paradigm affords the opportunity to tap semantic processing and lexical retrieval while manipulating and keeping under control the main language-specific features, e.g., morphology (nominal and verbal inflection) and syntax (determiner + noun agreement; subject + verb agreement). A third task should be dedicated either to specific properties of the languages spoken by the patients (e.g., relevant differences in the orthography) or to other language abilities (e.g., language switching or cross-linguistic translation) relevant for the linguistic needs and quality of life of patients (e.g., simultaneous translators, people living in multilingual countries like the Basque Country, Singapore, Switzerland, Italian autonomous provinces). When possible, the materials to be used in the intraoperative assessment of a multilingual patient should be tested on control groups composed of healthy speakers from the same environment (family members, friends, and multilingual speakers with similar linguistic profiles) in which reaction times and response accuracy are in principle roughly comparable with those of the patient. A summary of recommended perioperative assessment procedures to be used in awake neurosurgery settings with multilingual patients is provided in Table 7.

**TABLE 7:** Recommended perioperative assessment procedures in multilingual awake neurosurgery settings.

Experience-related linguistic factors	Variables to be assessed	Perioperative assessment	Recommendations
	AoA of each language		To perform an exhaustive neuropsychological examination
Multilingual profile	Setting where each language was acquired/learned	General preoperative assessment	To use standardized tests in the language most frequently used by the patient at the moment of surgery
	Primary language used in school education		
	Formal education received in each language		To obtain qualitative information about the patient’s performance
	Global amount of exposure to each language		
	Context Modality		To match the stimuli used in each language for length, phonological complexity, frequency, AoA, imageability, grammatical class, semantic category, syntactic features, morphological structure
	Language-related media preferences		Do not include culturally biased stimuli
Use of each language	Linguistic profile of interlocutors	Preoperative language assessment	To use appropriate semantic and phonological foils in comprehension tasks
	Frequency of use of each language in each modality in recent months		To provide clear and objective data about the distance and similarities between the languages spoken by the patient To provide assessment tests for the language-specific properties
	Context Modality Domain Perceived accent in each language		To test all languages To use a naming task with nouns (objects) and verbs (actions) inserted in a minimal phrasal context (”*This is the*…*; He/she/it*…”)
			To use an additional task dedicated to specific properties of the languages spoken by the patient or to language abilities relevant for his/her linguistic needs and quality of life (switching, translation, writing, reading)
Proficiency	Probability of spontaneous language switching	Intraoperative testing	
	Cross-linguistic flexibility		
	Amount of engagement in translation activity		
	Skills associated with effective communication in each language Family/friends and patient’s sense of impairment in each language		To test the selected materials and tasks on control groups of healthy speakers from the same linguistic environment of the patient

## Concluding Remarks

There is a critical need for a structured, theory-driven and evidence-based approach to multilingual patients in several clinical settings (neurological, neuropsychological, psychological, neurosurgical, and rehabilitative) since the number of people who use more than one language in everyday life is steadily increasing. For multilingual patients with tumors in language areas, awake brain surgery is used ever more often, as it allows maximizing the extent of resection while minimizing the functional risk.

However, this review shows that there is no consensus on the rationale that should underlie the selection of the neuropsychological tests to be included in the preoperative clinical work-up, and of the language paradigms to be used during language mapping procedures. The patient-tailored approach for perioperative assessment is still, necessarily, the preferred method due to the impossibility to predict all the combinations of languages spoken by multilinguals and to the difficulty in matching language-specific properties. The lack of such criteria may have serious implications and weaken the potential clinical benefit of awake surgery. For example, it could induce biases in deciding whether the outcome of intraoperative stimulation is due to interference with linguistic knowledge shared by all languages or specific for the language tested during stimulation. Moreover, given that neuroanatomical findings are highly inconsistent across studies, the present review highlights that the outcome of the neurosurgical procedure relies on an accurate planning of preoperative and intraoperative testing. Especially, this review illustrates the relevance of an objective and accurate description of both the linguistic profile of multilingual patients and the specific properties of the languages under scrutiny.

## Author Contributions

MD, AT, and GM contributed to conception and design of the study. MD and AM collected the data. MD, RC, and GM analyzed and interpreted the data. MD wrote the first draft of the manuscript. GM revised the text. GM and AT supervised the study. All authors discussed the results, contributed, and approved the final version of the manuscript.

## Conflict of Interest

The authors declare that the research was conducted in the absence of any commercial or financial relationships that could be construed as a potential conflict of interest.

## Publisher’s Note

All claims expressed in this article are solely those of the authors and do not necessarily represent those of their affiliated organizations, or those of the publisher, the editors and the reviewers. Any product that may be evaluated in this article, or claim that may be made by its manufacturer, is not guaranteed or endorsed by the publisher.

## Footnotes

1 For the sake of simplicity, in this manuscript we consider bilingualism (and literature on bilingualism) as an instance of multilingualism.

2 In this manuscript the term *language-specific* designates mechanisms and properties associated with each language spoken by a multilingual individual.

3 Data about monolingual patients or about patients with brain pathologies other than tumors, although they were included in the selected manuscripts, were not considered in this review.

4 Cognate words are pairs that are phonologically similar in the translation from one language to another (e.g., *origin* in English and *origine*, in Italian).

5 An extensive bilingual battery that provides more than 30 tasks matched across various language combinations is available for the evaluation of aphasic disorders: BAT (Bilingual Aphasia Test, https://www.mcgill.ca/linguistics/research/bat). When the language combination of a brain tumor patient corresponds to those included in the BAT (e.g., Italian-Slovenian or Italian-Spanish), its tests could be adapted and profitably used.

6 Involuntary language-switching phenomena induced by electrocortical stimulation are described in Tomasino et al. (2014).

7 Several databases can be used to obtain distributional measures in different languages (a list of some existing materials can be found at http://crr.ugent.be/archives/2141).
